# 4-(4-Chloro­phenyl­sulfan­yl)-1-[(*E*)-2-(4-chloro­phenyl­sulfan­yl)-1-phenyl­ethen­yl]-3-phenyl-1*H*-pyrazole

**DOI:** 10.1107/S1600536808031231

**Published:** 2008-10-04

**Authors:** P. Ramesh, A. Subbiahpandi, Ramaiyan Manikannan, S. Muthusubramanian, M. N. Ponnuswamy

**Affiliations:** a Department of Physics, Presidency College (Autonomous), Chennai 600 005, India; b Department of Organic Chemistry, School of Chemistry, Madurai Kamaraj University, Madurai 625 021, India; c Centre of Advanced Study in Crystallography and Biophysics, University of Madras, Guindy Campus, Chennai 600 025, India

## Abstract

In the title compound, C_29_H_20_Cl_2_N_2_S_2_, the pyrazole ring adopts a planar conformation. The chlorophenyl rings are twisted from the pyrazole ring at angles of 52.74 (14) and 29.92 (13)°, respectively. The crystal structure is stabilized by C—H⋯N and C—H⋯π inter­actions.

## Related literature

For the pharmacological and medicinal properties of the title compound, see: Baraldi *et al.* (1998 ▶); Bruno *et al.* (1990 ▶); Cottineau *et al.* (2002 ▶); Londershausen (1996 ▶); Chen & Li (1998 ▶); Mishra *et al.* (1998 ▶); Smith *et al.* (2001 ▶). For hybridization, see: Beddoes *et al.* (1986 ▶). For a related structure, see: Jin *et al.* (2004 ▶).
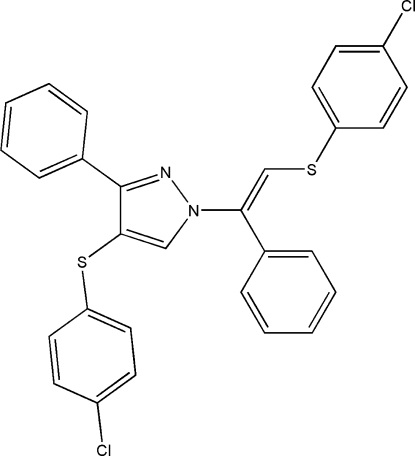


## Experimental

### 

#### Crystal data

C_29_H_20_Cl_2_N_2_S_2_
*M* *_r_* = 531.49
Monoclinic, 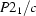

*a* = 12.3808 (4) Å
*b* = 21.4667 (7) Å
*c* = 10.4281 (4) Å
β = 108.181 (2)°
*V* = 2633.16 (16) Å^3^
*Z* = 4
Mo *K*α radiation
μ = 0.43 mm^−1^
*T* = 293 (2) K
0.30 × 0.20 × 0.18 mm

#### Data collection

Bruker Kappa APEXII diffractometer
Absorption correction: multi-scan (*SADABS*; Sheldrick, 2001 ▶) *T*_min_ = 0.883, *T*_max_ = 0.927
31420 measured reflections
6594 independent reflections
4292 reflections with *I* > 2σ(*I*)
*R*_int_ = 0.027

#### Refinement

*R*[*F* ^2^ > 2σ(*F* ^2^)] = 0.050
*wR*(*F* ^2^) = 0.142
*S* = 1.02
6594 reflections
316 parameters
6 restraints
H-atom parameters constrained
Δρ_max_ = 0.58 e Å^−3^
Δρ_min_ = −0.56 e Å^−3^

### 

Data collection: *APEX2* (Bruker, 2004 ▶); cell refinement: *APEX2*; data reduction: *SAINT* (Bruker, 2004 ▶); program(s) used to solve structure: *SHELXS97* (Sheldrick, 2008 ▶); program(s) used to refine structure: *SHELXL97* (Sheldrick, 2008 ▶); molecular graphics: *ORTEP-3* (Farrugia, 1997 ▶); software used to prepare material for publication: *SHELXL97* and *PLATON* (Spek, 2003 ▶).

## Supplementary Material

Crystal structure: contains datablocks global, I. DOI: 10.1107/S1600536808031231/bt2789sup1.cif

Structure factors: contains datablocks I. DOI: 10.1107/S1600536808031231/bt2789Isup2.hkl

Additional supplementary materials:  crystallographic information; 3D view; checkCIF report

## Figures and Tables

**Table 1:** Hydrogen-bond geometry (Å, °) *Cg* is the centroid of the C26–C31 ring.

*D*—H⋯*A*	*D*—H	H⋯*A*	*D*⋯*A*	*D*—H⋯*A*
C7—H7⋯N1	0.93	2.44	2.772 (3)	101
C21—H21⋯*Cg*^i^	0.93	2.96	3.808 (3)	153

Symmetry code: (i) 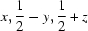.

**Fig. 1.**
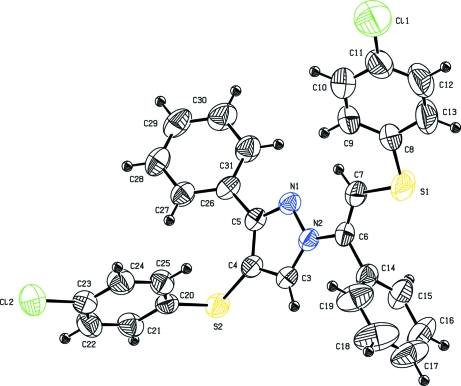
Molecular structure of the title compound, showing the atomic numbering and 50% probability displacement ellipsoids.

## supplementary crystallographic information

### Comment

Pyrazole derivatives possess significant antiarrhythmic and sedative (Bruno *et al.*, 1990), hypoglycemic (Cottineau *et al.*, 2002), antiviral (Baraldi *et al.*, 1998), and pesticidal (Londershausen *et al.*, 1996) properties. Some pyrazole derivatives are successfully tested for their antifungal (Chen & Li, 1998), antihistaminic (Mishra *et al.*, 1998) and anti-inflammatory (Smith *et al.*, 2001) activities.

An *ORTEP* plot of the molecule is shown in Fig. 1. The pyrazole ring adopts a planar conformation. The sum of the bond angles at N2 of the pyrazole ring (359.34°) is in accordance with *sp*^2^ hybridization (Beddoes *et al.*, 1986). The C—N bond lengths in the pyrazole ring are 1.340 (3) and 1.325 (3) Å, which are shorter than a C—N single bond length of 1.443 Å, but longer than a double bond length of 1.269 Å (Jin *et al.*, 2004), indicating electron delocalization. The chlorophenyl rings are twisted from the pyrazole ring at angles of 52.74 (14)° and 29.92 (13), respectively. The crystal packing shows weak C—H···N (Tab. 1) and C—H···π  interactions [C21-H21···cog^i^(C26,C27,C28,C29,C30,C31); symmetry operator (i) x, 1/2-y, 1/2+z: H···cog 2.956Å, C21···cog 3.808Å, C21-H21···cog 152.9°] in addition to van der Waals forces.

### Experimental

To a mixture of 2-[(4-chlorophenyl)sulfanyl]-1-phenyl-1-ethanone N-(E)-2- [(4-chlorophenyl)sulfanyl]-1-phenylethylidenehydrazone (0.003 mole) and 3 ml of dimethyl formamide kept in ice bath at 0° C, phosphorus oxycholride (0.024 mole) was added dropwise for 5–10 minutes. The reaction mixture was then kept in a microwave oven at 600 W for 30–60 sec. The process of the reaction was monitored by TLC. After completion of the reaction, the reaction mixture was poured into crushed ice and extracted with dichloromethane. The organic layer was dried in anhydrous sodium sulfate. The different compounds present in the mixture were separated by column chromatography using petroleum ether and ethyl acetate mixture as eluent. This isolated compound was recrystallized in dichloromethane to obtain 4-[(4-chlorophenyl)sulfanyl]-1- (*E*)-2-[(4-chlorophenyl)sulfanyl]-1-phenylethenyl-3-phenyl-1*H*-pyrazole in 86% yield.

### Refinement

All H atoms were positioned geometrically (C—H = 0.93 Å) and allowed to ride on their parent atoms, with *U*_iso_(H) = 1.2*U*_eq_(C) for all H atoms. The  atom S1 was restrained within an effective standard deviation of 0.1 so that their U_ij_ components approximate to isotropic bahavior.

### Crystal data

**Table:**

C_29_H_20_Cl_2_N_2_S_2_	*F*(000) = 1096
*M**_r_* = 531.49	*D*_x_ = 1.341 Mg m^−^^3^
Monoclinic, *P*2_1_/*c*	Mo *K*α radiation, λ = 0.71073 Å
Hall symbol: -P 2ybc	Cell parameters from 4538 reflections
*a* = 12.3808 (4) Å	θ = 1.9–28.4°
*b* = 21.4667 (7) Å	µ = 0.43 mm^−^^1^
*c* = 10.4281 (4) Å	*T* = 293 K
β = 108.181 (2)°	Block, colorless
*V* = 2633.16 (16) Å^3^	0.30 × 0.20 × 0.18 mm
*Z* = 4	

### Data collection

**Table:**

Bruker Kappa APEXII diffractometer	6594 independent reflections
Radiation source: fine-focus sealed tube	4292 reflections with *I* > 2σ(*I*)
graphite	*R*_int_ = 0.027
ω and φ scans	θ_max_ = 28.4°, θ_min_ = 1.9°
Absorption correction: multi-scan (SADABS; Sheldrick, 2001)	*h* = −9→16
*T*_min_ = 0.883, *T*_max_ = 0.927	*k* = −28→28
31420 measured reflections	*l* = −13→13

### Refinement

**Table:**

Refinement on *F*^2^	Primary atom site location: structure-invariant direct methods
Least-squares matrix: full	Secondary atom site location: difference Fourier map
*R*[*F*^2^ > 2σ(*F*^2^)] = 0.050	Hydrogen site location: inferred from neighbouring sites
*wR*(*F*^2^) = 0.142	H-atom parameters constrained
*S* = 1.02	*w* = 1/[σ^2^(*F*_o_^2^) + (0.0499*P*)^2^ + 1.5769*P*] where *P* = (*F*_o_^2^ + 2*F*_c_^2^)/3
6594 reflections	(Δ/σ)_max_ = 0.001
316 parameters	Δρ_max_ = 0.58 e Å^−^^3^
6 restraints	Δρ_min_ = −0.56 e Å^−^^3^

### Special details

**Table:**

Geometry. All e.s.d.'s (except the e.s.d. in the dihedral angle between two l.s. planes) are estimated using the full covariance matrix. The cell e.s.d.'s are taken into account individually in the estimation of e.s.d.'s in distances, angles and torsion angles; correlations between e.s.d.'s in cell parameters are only used when they are defined by crystal symmetry. An approximate (isotropic) treatment of cell e.s.d.'s is used for estimating e.s.d.'s involving l.s. planes.
Refinement. Refinement of *F*^2^ against ALL reflections. The weighted *R*-factor *wR* and goodness of fit *S* are based on *F*^2^, conventional *R*-factors *R* are based on *F*, with *F* set to zero for negative *F*^2^. The threshold expression of *F*^2^ > σ(*F*^2^) is used only for calculating *R*-factors(gt) *etc*. and is not relevant to the choice of reflections for refinement. *R*-factors based on *F*^2^ are statistically about twice as large as those based on *F*, and *R*- factors based on ALL data will be even larger.

### Fractional atomic coordinates and isotropic or equivalent isotropic displacement parameters (Å^2^)

**Table:**

	*x*	*y*	*z*	*U*_iso_*/*U*_eq_	
Cl1	−0.05360 (11)	0.97426 (6)	1.29295 (10)	0.1304 (4)	
Cl2	0.49992 (8)	0.55063 (4)	0.67733 (9)	0.0936 (3)	
S1	−0.22821 (6)	0.89305 (5)	0.68804 (10)	0.0988 (3)	
S2	0.13985 (5)	0.72882 (3)	0.29751 (6)	0.05543 (17)	
N2	−0.03047 (14)	0.82027 (8)	0.48954 (18)	0.0442 (4)	
N1	0.06227 (14)	0.85803 (8)	0.52541 (18)	0.0458 (4)	
C4	0.08940 (18)	0.77699 (10)	0.4007 (2)	0.0457 (5)	
C5	0.13504 (17)	0.83241 (10)	0.4710 (2)	0.0432 (5)	
C3	−0.01665 (18)	0.77170 (10)	0.4154 (2)	0.0474 (5)	
H3	−0.0692	0.7402	0.3804	0.057*	
C6	−0.12269 (18)	0.83142 (11)	0.5402 (2)	0.0487 (5)	
C7	−0.1128 (2)	0.87324 (13)	0.6357 (3)	0.0660 (7)	
H7	−0.0430	0.8924	0.6756	0.079*	
C8	−0.1672 (2)	0.91670 (13)	0.8564 (3)	0.0708 (7)	
C9	−0.0600 (2)	0.90247 (14)	0.9387 (3)	0.0749 (8)	
H9	−0.0108	0.8804	0.9040	0.090*	
C10	−0.0243 (3)	0.92044 (15)	1.0718 (4)	0.0844 (9)	
H10	0.0490	0.9110	1.1262	0.101*	
C11	−0.0963 (3)	0.95220 (14)	1.1244 (3)	0.0815 (9)	
C12	−0.2020 (3)	0.96751 (19)	1.0443 (4)	0.1049 (12)	
H12	−0.2504	0.9899	1.0797	0.126*	
C13	−0.2377 (3)	0.95004 (19)	0.9112 (4)	0.1074 (13)	
H13	−0.3104	0.9608	0.8570	0.129*	
C14	−0.22593 (18)	0.79358 (11)	0.4766 (2)	0.0521 (5)	
C15	−0.3037 (2)	0.81280 (15)	0.3589 (3)	0.0748 (8)	
H15	−0.2918	0.8497	0.3186	0.090*	
C16	−0.4000 (3)	0.7778 (2)	0.2995 (4)	0.0977 (11)	
H16	−0.4524	0.7911	0.2192	0.117*	
C17	−0.4182 (3)	0.7247 (2)	0.3572 (4)	0.1013 (13)	
H17	−0.4834	0.7014	0.3171	0.122*	
C18	−0.3416 (4)	0.7046 (2)	0.4741 (4)	0.1147 (15)	
H18	−0.3544	0.6677	0.5137	0.138*	
C19	−0.2445 (3)	0.73921 (16)	0.5344 (3)	0.0901 (10)	
H19	−0.1919	0.7254	0.6141	0.108*	
C20	0.23637 (18)	0.67789 (10)	0.4109 (2)	0.0465 (5)	
C21	0.3060 (2)	0.64266 (12)	0.3584 (3)	0.0584 (6)	
H21	0.2985	0.6456	0.2670	0.070*	
C22	0.3863 (2)	0.60336 (12)	0.4394 (3)	0.0656 (7)	
H22	0.4333	0.5801	0.4035	0.079*	
C23	0.3961 (2)	0.59894 (11)	0.5732 (3)	0.0615 (6)	
C24	0.3265 (2)	0.63248 (13)	0.6277 (3)	0.0653 (7)	
H24	0.3333	0.6286	0.7187	0.078*	
C25	0.2463 (2)	0.67213 (12)	0.5457 (2)	0.0571 (6)	
H25	0.1989	0.6950	0.5817	0.069*	
C26	0.24546 (18)	0.86348 (11)	0.4912 (2)	0.0471 (5)	
C27	0.34364 (19)	0.83066 (12)	0.5004 (2)	0.0544 (6)	
H27	0.3402	0.7878	0.4864	0.065*	
C28	0.4473 (2)	0.86127 (14)	0.5304 (3)	0.0653 (7)	
H28	0.5130	0.8389	0.5364	0.078*	
C29	0.4531 (2)	0.92423 (15)	0.5512 (3)	0.0738 (8)	
H29	0.5228	0.9445	0.5725	0.089*	
C30	0.3564 (2)	0.95734 (14)	0.5405 (3)	0.0780 (8)	
H30	0.3604	1.0003	0.5534	0.094*	
C31	0.2524 (2)	0.92733 (12)	0.5107 (3)	0.0637 (7)	
H31	0.1870	0.9502	0.5039	0.076*	

### Atomic displacement parameters (Å^2^)

**Table:**

	*U*^11^	*U*^22^	*U*^33^	*U*^12^	*U*^13^	*U*^23^
Cl1	0.1506 (10)	0.1492 (10)	0.0782 (6)	0.0227 (8)	0.0167 (6)	−0.0108 (6)
Cl2	0.0875 (6)	0.0736 (5)	0.1054 (6)	0.0208 (4)	0.0094 (5)	0.0060 (4)
S1	0.0426 (4)	0.1458 (8)	0.1049 (6)	0.0127 (4)	0.0184 (4)	−0.0526 (6)
S2	0.0496 (3)	0.0730 (4)	0.0426 (3)	0.0013 (3)	0.0128 (2)	−0.0085 (3)
N2	0.0340 (9)	0.0478 (10)	0.0491 (10)	−0.0018 (7)	0.0103 (7)	0.0014 (8)
N1	0.0401 (9)	0.0447 (9)	0.0511 (10)	−0.0051 (7)	0.0119 (8)	0.0017 (8)
C4	0.0388 (11)	0.0537 (12)	0.0425 (11)	−0.0019 (9)	0.0097 (9)	−0.0018 (9)
C5	0.0382 (11)	0.0498 (12)	0.0404 (10)	−0.0024 (9)	0.0103 (8)	0.0055 (9)
C3	0.0373 (11)	0.0513 (12)	0.0504 (12)	−0.0036 (9)	0.0089 (9)	−0.0035 (10)
C6	0.0351 (11)	0.0543 (13)	0.0546 (13)	0.0051 (9)	0.0109 (9)	0.0013 (10)
C7	0.0375 (12)	0.0774 (17)	0.0822 (18)	0.0019 (11)	0.0173 (12)	−0.0179 (15)
C8	0.0533 (15)	0.0706 (17)	0.089 (2)	0.0111 (13)	0.0222 (14)	−0.0161 (15)
C9	0.0548 (16)	0.0710 (18)	0.099 (2)	0.0148 (13)	0.0241 (15)	−0.0101 (16)
C10	0.0685 (19)	0.084 (2)	0.092 (2)	0.0161 (16)	0.0113 (16)	0.0015 (18)
C11	0.091 (2)	0.0686 (18)	0.080 (2)	0.0153 (16)	0.0203 (17)	−0.0020 (15)
C12	0.103 (3)	0.118 (3)	0.092 (2)	0.049 (2)	0.028 (2)	−0.021 (2)
C13	0.074 (2)	0.142 (3)	0.098 (3)	0.051 (2)	0.0150 (18)	−0.031 (2)
C14	0.0384 (11)	0.0631 (14)	0.0565 (13)	−0.0014 (10)	0.0175 (10)	−0.0032 (11)
C15	0.0466 (14)	0.087 (2)	0.0811 (19)	−0.0006 (13)	0.0055 (13)	0.0086 (16)
C16	0.0480 (16)	0.140 (3)	0.090 (2)	−0.0098 (19)	−0.0007 (15)	−0.009 (2)
C17	0.072 (2)	0.147 (4)	0.092 (3)	−0.055 (2)	0.0362 (19)	−0.042 (2)
C18	0.135 (4)	0.118 (3)	0.096 (3)	−0.071 (3)	0.043 (3)	−0.011 (2)
C19	0.099 (2)	0.091 (2)	0.0697 (19)	−0.0358 (19)	0.0118 (17)	0.0075 (17)
C20	0.0432 (11)	0.0498 (12)	0.0489 (12)	−0.0083 (9)	0.0177 (9)	−0.0106 (10)
C21	0.0645 (15)	0.0629 (15)	0.0545 (14)	−0.0016 (12)	0.0283 (12)	−0.0093 (12)
C22	0.0661 (16)	0.0593 (15)	0.0797 (18)	0.0048 (13)	0.0347 (14)	−0.0102 (13)
C23	0.0583 (15)	0.0484 (13)	0.0731 (17)	−0.0009 (11)	0.0136 (13)	−0.0042 (12)
C24	0.0750 (18)	0.0684 (16)	0.0510 (14)	0.0034 (14)	0.0175 (12)	−0.0034 (12)
C25	0.0569 (14)	0.0674 (15)	0.0510 (13)	0.0055 (12)	0.0227 (11)	−0.0067 (11)
C26	0.0421 (11)	0.0585 (13)	0.0391 (11)	−0.0097 (10)	0.0103 (9)	0.0051 (9)
C27	0.0453 (12)	0.0629 (14)	0.0555 (13)	−0.0062 (11)	0.0164 (10)	−0.0006 (11)
C28	0.0421 (13)	0.0853 (19)	0.0682 (16)	−0.0075 (12)	0.0167 (11)	0.0051 (14)
C29	0.0478 (15)	0.085 (2)	0.0841 (19)	−0.0233 (14)	0.0140 (13)	0.0101 (16)
C30	0.0644 (18)	0.0612 (16)	0.102 (2)	−0.0207 (14)	0.0169 (16)	0.0073 (15)
C31	0.0503 (14)	0.0570 (15)	0.0802 (18)	−0.0086 (11)	0.0150 (12)	0.0087 (13)

### Geometric parameters (Å, °)

**Table:**

Cl1—C11	1.735 (3)	C15—H15	0.9300
Cl2—C23	1.742 (3)	C16—C17	1.340 (5)
S1—C7	1.734 (3)	C16—H16	0.9300
S1—C8	1.756 (3)	C17—C18	1.360 (6)
S2—C4	1.742 (2)	C17—H17	0.9300
S2—C20	1.772 (2)	C18—C19	1.386 (5)
N2—C3	1.340 (3)	C18—H18	0.9300
N2—N1	1.359 (2)	C19—H19	0.9300
N2—C6	1.420 (3)	C20—C25	1.378 (3)
N1—C5	1.325 (3)	C20—C21	1.382 (3)
C4—C3	1.373 (3)	C21—C22	1.375 (4)
C4—C5	1.419 (3)	C21—H21	0.9300
C5—C26	1.476 (3)	C22—C23	1.366 (4)
C3—H3	0.9300	C22—H22	0.9300
C6—C7	1.318 (3)	C23—C24	1.374 (4)
C6—C14	1.485 (3)	C24—C25	1.383 (4)
C7—H7	0.9300	C24—H24	0.9300
C8—C9	1.371 (4)	C25—H25	0.9300
C8—C13	1.383 (4)	C26—C27	1.382 (3)
C9—C10	1.374 (4)	C26—C31	1.384 (3)
C9—H9	0.9300	C27—C28	1.388 (3)
C10—C11	1.366 (4)	C27—H27	0.9300
C10—H10	0.9300	C28—C29	1.367 (4)
C11—C12	1.355 (5)	C28—H28	0.9300
C12—C13	1.371 (5)	C29—C30	1.367 (4)
C12—H12	0.9300	C29—H29	0.9300
C13—H13	0.9300	C30—C31	1.386 (3)
C14—C19	1.365 (4)	C30—H30	0.9300
C14—C15	1.366 (4)	C31—H31	0.9300
C15—C16	1.382 (4)		
			
C7—S1—C8	104.26 (13)	C15—C16—H16	119.9
C4—S2—C20	104.53 (10)	C16—C17—C18	120.2 (3)
C3—N2—N1	111.96 (17)	C16—C17—H17	119.9
C3—N2—C6	127.43 (18)	C18—C17—H17	119.9
N1—N2—C6	120.35 (17)	C17—C18—C19	120.0 (4)
C5—N1—N2	105.27 (17)	C17—C18—H18	120.0
C3—C4—C5	104.59 (19)	C19—C18—H18	120.0
C3—C4—S2	124.02 (17)	C14—C19—C18	120.0 (3)
C5—C4—S2	130.90 (16)	C14—C19—H19	120.0
N1—C5—C4	110.79 (18)	C18—C19—H19	120.0
N1—C5—C26	118.32 (19)	C25—C20—C21	119.2 (2)
C4—C5—C26	130.9 (2)	C25—C20—S2	124.29 (17)
N2—C3—C4	107.39 (19)	C21—C20—S2	116.56 (18)
N2—C3—H3	126.3	C22—C21—C20	120.9 (2)
C4—C3—H3	126.3	C22—C21—H21	119.6
C7—C6—N2	120.0 (2)	C20—C21—H21	119.6
C7—C6—C14	125.0 (2)	C23—C22—C21	119.2 (2)
N2—C6—C14	114.93 (19)	C23—C22—H22	120.4
C6—C7—S1	120.9 (2)	C21—C22—H22	120.4
C6—C7—H7	119.5	C22—C23—C24	121.3 (2)
S1—C7—H7	119.5	C22—C23—Cl2	119.4 (2)
C9—C8—C13	117.9 (3)	C24—C23—Cl2	119.3 (2)
C9—C8—S1	126.3 (2)	C23—C24—C25	119.2 (2)
C13—C8—S1	115.7 (2)	C23—C24—H24	120.4
C8—C9—C10	120.9 (3)	C25—C24—H24	120.4
C8—C9—H9	119.6	C20—C25—C24	120.3 (2)
C10—C9—H9	119.6	C20—C25—H25	119.8
C11—C10—C9	120.1 (3)	C24—C25—H25	119.8
C11—C10—H10	120.0	C27—C26—C31	118.8 (2)
C9—C10—H10	120.0	C27—C26—C5	122.3 (2)
C12—C11—C10	119.9 (3)	C31—C26—C5	118.7 (2)
C12—C11—Cl1	119.3 (3)	C26—C27—C28	120.4 (2)
C10—C11—Cl1	120.8 (3)	C26—C27—H27	119.8
C11—C12—C13	120.1 (3)	C28—C27—H27	119.8
C11—C12—H12	119.9	C29—C28—C27	120.2 (3)
C13—C12—H12	119.9	C29—C28—H28	119.9
C12—C13—C8	121.0 (3)	C27—C28—H28	119.9
C12—C13—H13	119.5	C30—C29—C28	120.0 (2)
C8—C13—H13	119.5	C30—C29—H29	120.0
C19—C14—C15	119.1 (3)	C28—C29—H29	120.0
C19—C14—C6	120.7 (2)	C29—C30—C31	120.4 (3)
C15—C14—C6	120.2 (2)	C29—C30—H30	119.8
C14—C15—C16	120.4 (3)	C31—C30—H30	119.8
C14—C15—H15	119.8	C26—C31—C30	120.2 (3)
C16—C15—H15	119.8	C26—C31—H31	119.9
C17—C16—C15	120.2 (3)	C30—C31—H31	119.9
C17—C16—H16	119.9		
			
C3—N2—N1—C5	0.5 (2)	C7—C6—C14—C15	94.6 (3)
C6—N2—N1—C5	175.07 (18)	N2—C6—C14—C15	−84.7 (3)
C20—S2—C4—C3	−104.7 (2)	C19—C14—C15—C16	0.2 (5)
C20—S2—C4—C5	84.6 (2)	C6—C14—C15—C16	−179.9 (3)
N2—N1—C5—C4	−0.8 (2)	C14—C15—C16—C17	0.3 (5)
N2—N1—C5—C26	179.40 (17)	C15—C16—C17—C18	−0.5 (6)
C3—C4—C5—N1	0.8 (2)	C16—C17—C18—C19	0.1 (7)
S2—C4—C5—N1	172.85 (17)	C15—C14—C19—C18	−0.5 (5)
C3—C4—C5—C26	−179.5 (2)	C6—C14—C19—C18	179.6 (3)
S2—C4—C5—C26	−7.4 (4)	C17—C18—C19—C14	0.3 (6)
N1—N2—C3—C4	−0.1 (2)	C4—S2—C20—C25	11.8 (2)
C6—N2—C3—C4	−174.1 (2)	C4—S2—C20—C21	−167.52 (18)
C5—C4—C3—N2	−0.4 (2)	C25—C20—C21—C22	−1.6 (4)
S2—C4—C3—N2	−173.19 (16)	S2—C20—C21—C22	177.8 (2)
C3—N2—C6—C7	164.7 (2)	C20—C21—C22—C23	0.6 (4)
N1—N2—C6—C7	−8.9 (3)	C21—C22—C23—C24	0.7 (4)
C3—N2—C6—C14	−15.9 (3)	C21—C22—C23—Cl2	−178.5 (2)
N1—N2—C6—C14	170.50 (18)	C22—C23—C24—C25	−1.0 (4)
N2—C6—C7—S1	173.84 (18)	Cl2—C23—C24—C25	178.2 (2)
C14—C6—C7—S1	−5.5 (4)	C21—C20—C25—C24	1.2 (4)
C8—S1—C7—C6	152.2 (2)	S2—C20—C25—C24	−178.1 (2)
C7—S1—C8—C9	−19.9 (3)	C23—C24—C25—C20	0.0 (4)
C7—S1—C8—C13	163.8 (3)	N1—C5—C26—C27	147.7 (2)
C13—C8—C9—C10	0.5 (5)	C4—C5—C26—C27	−32.0 (3)
S1—C8—C9—C10	−175.8 (3)	N1—C5—C26—C31	−27.8 (3)
C8—C9—C10—C11	0.9 (5)	C4—C5—C26—C31	152.5 (2)
C9—C10—C11—C12	−1.8 (6)	C31—C26—C27—C28	0.8 (3)
C9—C10—C11—Cl1	179.0 (3)	C5—C26—C27—C28	−174.7 (2)
C10—C11—C12—C13	1.4 (6)	C26—C27—C28—C29	0.0 (4)
Cl1—C11—C12—C13	−179.4 (3)	C27—C28—C29—C30	−0.9 (4)
C11—C12—C13—C8	0.0 (7)	C28—C29—C30—C31	1.0 (5)
C9—C8—C13—C12	−1.0 (6)	C27—C26—C31—C30	−0.7 (4)
S1—C8—C13—C12	175.7 (4)	C5—C26—C31—C30	174.9 (2)
C7—C6—C14—C19	−85.5 (4)	C29—C30—C31—C26	−0.1 (5)
N2—C6—C14—C19	95.2 (3)		

### Hydrogen-bond geometry (Å, °)

**Table:**

*D*—H···*A*	*D*—H	H···*A*	*D*···*A*	*D*—H···*A*
C7—H7···N1	0.93	2.44	2.772 (3)	101
